# Change in level of physical activity during pregnancy in obese women: findings from the UPBEAT pilot trial

**DOI:** 10.1186/s12884-015-0479-2

**Published:** 2015-03-01

**Authors:** Louise Hayes, Catherine Mcparlin, Tarja I Kinnunen, Lucilla Poston, Stephen C Robson, Ruth Bell, On behalf of the UPBEAT Consortium

**Affiliations:** Institute of Health & Society, Newcastle University, Baddiley-Clark Building, Richardson Road, Newcastle upon Tyne, NE2 4AX UK; Institute of Cellular Medicine, Newcastle University, Newcastle upon Tyne, UK; Newcastle-upon-Tyne Hospitals NHS Foundation Trust, Newcastle upon Tyne, UK; School of Health Sciences, University of Tampere, Tampere, Finland; Division of Women’s Health, Women’s Health Academic Centre, King’s College, London, UK

**Keywords:** Maternal obesity, Accelerometer, MVPA, Socio-demographic factors

## Abstract

**Background:**

Maternal obesity is associated with an increased risk of pregnancy complications, including gestational diabetes. Physical activity (PA) might improve glucose metabolism and reduce the incidence of gestational diabetes. The purpose of this study was to explore patterns of PA and factors associated with change in PA in obese pregnant women.

**Methods:**

PA was assessed objectively by accelerometer at 16 – 18 weeks’ (T0), 27 – 28 weeks’ (T1) and 35 – 36 weeks’ gestation (T2) in 183 obese pregnant women recruited to a pilot randomised trial of a combined diet and PA intervention (the UPBEAT study).

**Results:**

Valid PA data were available for 140 (77%), 76 (42%) and 54 (30%) women at T0, T1 and T2 respectively. Moderate and vigorous physical activity as a proportion of accelerometer wear time declined with gestation from a median of 4.8% at T0 to 3% at T2 (p < 0.05). Total activity as a proportion of accelerometer wear time did not change. Being more active in early pregnancy was associated with a higher level of PA later in pregnancy. The intervention had no effect on PA.

**Conclusions:**

PA in early pregnancy was the factor most strongly associated with PA at later gestations. Women should be encouraged to participate in PA before becoming pregnant and to maintain their activity levels during pregnancy. There is a need for effective interventions, tailored to the needs of individuals and delivered early in pregnancy to support obese women to be sufficiently active during pregnancy.

**Trial registration:**

Current Controlled Trials ISRCTN89971375 (Registered 28/11/2008).

## Background

Gestational diabetes (GDM; defined as diabetes or impaired glucose tolerance that is first recognised during pregnancy [1]) is associated with maternal obesity [2]. Physical activity (PA) during pregnancy might reduce GDM risk. A meta-analysis reported a 24% reduction in GDM incidence among women (unselected for BMI status) who were active in early pregnancy compared to those who were inactive [3]. Current guidance for pregnant women recommends 30 minutes of daily moderate intensity PA [4,5].

Data on objectively measured PA during pregnancy are sparse but indicate that PA declines with gestation [6-8]. Data on obese pregnant women are even more limited but suggest a similar, or greater, decline in PA [9,10]. A recent systematic review concluded that more detailed description of PA in this population was needed [11].

Effective interventions that impact on GDM incidence by supporting obese pregnant women to be active are lacking [12,13]. A better understanding of factors influencing PA during pregnancy would help to inform the development of such interventions.

We aimed to describe objectively measured PA during pregnancy in obese women enrolled in the UK Pregnancies Better Eating and Activity (UPBEAT) pilot trial [14] and to explore factors associated with PA in these women.

## Methods

### The UPBEAT trial

UPBEAT aims to improve glycaemic control in obese women through a combined behaviour change intervention targeting PA and diet. (Current Controlled Trials ISRCTN89971375; registered 28/11/2008). A pilot study to determine the effect of the intervention on diet and PA behaviours was undertaken in one hundred and eighty-three obese (BMI ≥30 kg/m^2^) women, with a singleton pregnancy of 15 to 18 weeks’ gestation [14]. Women were recruited from four ante-natal clinics within the UK between March 2009 and May 2011, and were randomised to receive the intervention or standard care. The methods have been reported previously [14]. There was no statistically significant difference between the intervention and control groups in PA measured by accelerometry at baseline or follow-up in the UPBEAT pilot trial. Median (interquartile range) minutes per day active at baseline was 217.4 (171.3, 268.3) in women in the intervention group and 213.7 (167.7, 263.7) in the control group (p = 0.638). At first follow-up the figures were 188.1 (151.6, 244.2) and 199.2 (147.0, 237.9) respectively (p = 0.316) and at second follow-up 202.7 (178.6, 228.6) and 189.7 (130.3, 236.6) respectively (p = 0.455). As PA at baseline and follow-up was similar in women in the intervention and control groups, data from the intervention and control arms of the trial were combined for this study. This was a post-hoc decision, made after examination of the data.

### PA measurement

PA was measured using an Actigraph**™** accelerometer at 16^+0^ – 18^+6^ weeks’ gestation (T0), 27^+0^ – 28^+6^ weeks’ gestation (T1) and 35^+0^ – 36^+6^ weeks’ gestation (T2). The Actigraph is considered appropriate for use in pregnancy and has previously been used to measure PA in overweight and obese pregnant women [15,16]. Data were processed using Actilife software [17]. Freedson’s cut points were used to categorise time as sedentary (SED; <100 counts per minute (cpm)), light activity (LPA; 100–1951 cpm), and moderate or vigorous intensity activity (MVPA; >1951 cpm) [18]. All activity (ACTIVE; ≥100 cpm) was also calculated. Data from participants recording ≥3 days of valid (≥500 minutes per day) accelerometry were included in the analysis. PA data for each individual were summarised as median minutes per day in each intensity category. Change in time spent in different PA intensities was calculated as the difference in minutes per day recorded in each intensity (T1 –T0 and T2 –T0). As accelerometer wear time (valid minutes of data recorded) decreased from baseline to follow-up, PA of different intensities as a proportion of total wear time was also calculated (mins per day in each activity intensity/mins per day accelerometer worn). Previous work reports that total activity, rather than sub-components of activity, is most strongly associated with glucose homeostasis [19]. We therefore sought to identify factors associated with proportion of accelerometer wear time ≥100 cpm recorded (%ACTIVE). Women were categorised as recording above or below median %ACTIVE at each time point. They were further categorised as reducing %ACTIVE by greater than or less than the median at T1 (−1.5%) and T2 (−0.5%).

### Statistical analysis

Data analysis was performed using SPSS version 21.0. Variables were checked for normal distribution using the Shapiro-Wilk test. Descriptive statistics are presented as mean (SD), median (inter-quartile range) or proportions, as appropriate. Wilcoxon matched-pairs signed rank tests were used to assess change in PA. Logistic regression was used to explore associations of maternal characteristics with absolute duration of and change in PA.

The following variables were included in the analyses: BMI (kg/m^2^) at T0; age (years) at T0; parity (nulliparous or parous); smoking status (self-reported never or ex/current smoker at T0); ethnicity (White or non-White); marital status (married/cohabiting or single/divorced/separated); highest educational attainment (degree or higher or no degree); employment status (in paid employment or not in paid employment); living accommodation (owner occupier/private rented or council rented); Index of Multiple Deprivation (IMD; quintile 5 [most deprived] compared to quintile 1–4). All data were collected at baseline by the research midwife and entered immediately into the study database.

### Ethics

Research Ethics Committee approval was obtained in all participating centres (London, Newcastle and Glasgow), UK Integrated Research Application System; reference 09/H0802/5 (South East London Research Ethics Committee). Written informed consent for participation in the study was obtained from all participants.

## Results

One hundred and forty of 183 (77%) women recruited to the study provided sufficient PA data to be included at T0. Median BMI was 33.8 (IQR 31.9, 37.6) and median age was 32 years (IQR 26, 35 years) (Table 1).

**Table 1:** **Baseline characteristics of participants with valid accelerometry data at T0, T1 and T2**

	**T0**		**T1**		**T2**	
	**16** ^**+0**^ **– 18** ^**+6**^ **weeks’ gestation**		**27** ^**+0**^ **– 28** ^**+6**^ **weeks’ gestation**		**35** ^**+0**^ **– 36** ^**+6**^ **weeks’ gestation**	
≥3 days valid data (% consented)	140	(76.5)	76	(41.5)	54	(29.5)
BMI at T0 (Kg/m^2^)^1^	33.8	(31.9, 37.6)	34.1	(32.4, 37.1)	35.6	(33.4, 38.4)**
Age (years) ^1^	32	(26, 35)	32	(28, 35)	33	(28, 36)**
Parity						
*Nulliparous*	62	(45.9)	26	(34.7)*	17	(31.5)**
*Parous*	73	(54.1)	49	(65.3)	37	(68.5)
Smoking status						
*Current or ex-smoker*	46	(34.1)	30	(40.0)	20	(37.0)
*Never smoked*	89	(65.9)	45	(60.0)	34	(63.0)
Ethnicity						
*White*	84	(62.2)	48	(64.0)	34	(63.0)
*Black, Asian or other*	51	(37.8)	27	(36.0)	20	(37.0)
Marital status						
*Married/cohabiting*	69	(51.1)	38	(50.7)	28	(51.9)
*Single/divorced/separate*	66	(48.9)	37	(49.3)	26	(48.1)
Educational achievement						
*Degree or higher*	62	(45.9)	35	(46.7)	26	(48.1)
*No degree*	73	(54.1)	40	(53.3)	28	(51.9)
Employment status						
*Paid or self employment*	92	(68.7)	50	(66.7)	40	(74.1)
*Not in paid employment*	42	(31.3)	25	(33.3)	14	(25.9)
Living accommodation						
*Owned or private rented*	76	(54.3)	48	(63.2)*	36	(66.7)**
*Rented (council)*	64	(45.7)	28	(36.8)	18	(33.3)
IMD quintile						
1 *(least deprived)*	4	(3.4)	4	(6.2)	3	(6.5)
2	4	(3.4)	2	(3.1)	1	(2.2)
3	14	(12.1)	7	(10.8)	4	(8.7)
4	44	(37.9)	26	(40.0)	21	(45.7)
5 *(most deprived)*	50	(43.1)	26	(40.0)	17	(37.0)
Weight gain^2^						
*Above IOM guideline*					23	(45.1)
*Within or below IOM guideline*					28	(54.9)

Figures are n(%); ^1^Median (inter-quartile range); ^2^The American Institute of Medicine (IOM) recommends that obese women should gain between 5–9 kg during pregnancy.

*p < 0.05 for difference between baseline and 28 weeks.

**p < 0.05 for difference between baseline and 35 weeks.

At T1, 76 (42%) women and at T2, 54 (30%) women provided valid PA data. Women who provided valid accelerometry data at all 3 data collection points were similar to women who did not in degree of obesity (p = 0.092), ethnicity (p = 0.712), and IMD score (p = 0.604), but were older (33 years vs 32 years; p = 0.020), more likely to have at least one child (p = 0.001) and less likely to live in council rented accommodation (p = 0.004).

The number of minutes spent in SED, LPA, MVPA and ACTIVE was lower at T1 than at T0 (Table 2). At T2 time spent in SED, MVPA and ACTIVE was lower than at T0. MVPA also declined between T1 and T2. A decrease in total MVPA from a median (inter-quartile range) at T0 of 39 mins/day (25, 52) to 34.5 (24, 44) at T1 and 23 (18, 38) at T2 was recorded. MVPA as a proportion of wear time (%MVPA), but not %LPA or %ACTIVE, also decreased with gestation (Table 2).

**Table 2:** **Change between T0, T1 and T2 in sedentary time, LPA, MVPA and total time active**

	**T0**	**T1**	**Change**	**T2**	**Change**
	**(n = 140)**	**(n = 76)**	**(T1-T0)**	**(n = 54)**	**(T2-T0)**
***Absolute values*** **(min/day)**					
**SED**	576.5	555.1	−21.5*	571.8	−4.7*
	(510.6, 642.8)	(505.6, 635.3)		(507.5, 615.9)	
**LPA**	174.8	154.8	−20.0*	169.7	−5.1
	(140.3, 222.2)	(124.9, 191.7)		(142.7, 199.2)	
**MVPA**	39.0	34.5	−4.5*	23.3	−15.7*^ƚ^
	(24.7, 51.9)	(23.9, 43.5)		(18.0, 38.0)	
**ACTIVE**	215.6	194.8	−20.8*	198.6	−17.0*
	(168.2, 264.9)	(146.2, 228.3)		(163.4, 228.6)	
***Proportion of time accelerometer worn*** **(% worn time)**					
**%SED**	73.2	75.3	1.3	73.8	0.5
	(68.5, 78.4)	(70.5, 80.5)	(−2.5, 5.8)	(70.6, 79.9)	(−2.2, 4.1)
**%LPA**	21.5	20.1	−0.5	21.9	0.9
	(17.6, 25.8)	(16.3, 25.5)	(−4.1, 1.9)	(17.8, 25.4)	(−2.7, 2.8)
**%MVPA**	4.8	4.3	−0.4	3.0	−1.1*^ƚ^
	(3.1, 6.3)	(2.9, 5.2)	(−1.3, 0.6)	(2.3, 4.6)	(−2.5, −0.3)
**%ACTIVE**	26.8	24.7	−1.5	26.3	−0.5
	(21.6, 31.5)	(19.6, 29.5)	(−5.8, 2.7)	(20.1, 29.4)	(−4.1, 2.2)
**Mean counts per minute (SD)**	299	278	-	248*	-
	(114)	(119)		(93)	

Figures are median (IQR) or mean (SD); Wilcoxon Signed Ranks Test or Paired Samples t-test used to test for differences.

Definition of PA intensity: <100 cpm = Sedentary (SED); 100-1951 cpm = Light PA (LPA), >1951 cpm = Moderate + vigorous PA (MVPA); ≥100 cpm = total activity (ACTIVE).

*p < 0.05 for change in median or mean between T0 and T1 or T0 and T2.

^ƚ^p < 0.05 for change in median or mean between T1 and T2.

T0 = 16^+0^ – 18^+6^ weeks’ gestation; T1 = 27^+0^ – 28^+6^ weeks’ gestation; T2 = 35^+0^ – 36^+6^ weeks’ gestation.

Having at least one child (OR 2.73; 95% CI 1.36, 5.48) and not having a degree (OR 4.03; 95% CI 1.96, 8.28) were associated with greater than median %ACTIVE at T0 (Table 3). At T1 non-white ethnicity was associated with greater than median %ACTIVE (OR 3.96; 95% CI 1.44, 10.89). A recording greater than median %ACTIVE at T0 was strongly associated with %ACTIVE at T1 (OR 5.85; 2.16, 15.86) and T2 (OR 5.95; 1.80, 19.70). A recording greater than median %ACTIVE at T1 was strongly associated with %ACTIVE at T2 (OR 4.61; 1.39, 15.24).

**Table 3:** **Unadjusted odds ratio of recording more than median time ACTIVE at T0, T1 and T2 as proportion of accelerometer wear time, by baseline characteristics**

	**T0**		**T1**		**T2**	
	**OR**	**95% CI**	**OR**	**95% CI**	**OR**	**95% CI**
Intervention status						
*Intervention*	Ref		Ref		Ref	
*Control*	0.89	0.46, 1.73	0.81	0.33, 1.99	0.86	0.30, 2.51
Body Mass Index (Kg/m^2^)						
*30-34.9*	Ref		Ref		Ref	
*35+*	1.81	0.89, 3.66	0.85	0.34, 2.14	1.25	0.42, 3.70
Age (years)						
*<35*	Ref		Ref		Ref	
*≥35*	0.78	0.37, 1.67	1.40	0.52, 3.77	0.53	0.17, 1.62
Parity						
*Nulliparous*	Ref		Ref		Ref	
*Parous*	**2.73**	**1.36, 5.48**	1.04	0.40, 2.70	1.19	0. 38, 3.75
Smoking status						
*Current or ex-smoker*	1.46	0.71, 2.98	1.20	0.47, 3.01	0.53	0.17, 1.62
*Never smoked*	Ref		Ref		Ref	
Ethnicity						
*White*	Ref		Ref		Ref	
*Black, Asian or other*	1.09	0.54, 2.19	**3.96**	**1.44, 10.89**	0.53	0.17, 1.62
Marital status						
*Married/cohabiting*	Ref		Ref		Ref	
*Single/divorced/separate*	0.72	0.37, 1.42	1.17	0.47, 2.90	1.00	0.34, 2.91
Educational achievement						
*Degree or higher*	Ref		Ref		Ref	
*No degree*	**4.03**	**1.96, 8.28**	1.80	0.72, 4.51	1.82	0.62, 5.35
Employment status						
*Paid or self employment*	Ref		Ref		Ref	
*Not in paid employment*	1.46	0.70, 3.04	1.76	0.67, 4.67	2.20	0.63, 7.74
Living accommodation						
*Owned or private rented*	Ref		Ref		Ref	
*Rented (council)*	1.00	0.51, 1.95	1.00	0.39, 2.54	0.72	0.23, 2.23
IMD quintile						
*1-4*	Ref		Ref		Ref	
5 *(most deprived)*	1.53	0.73, 3.20	1.23	0.45, 3.32	1.05	0.32, 3.48
Baseline ACTIVE						
*Below median*	-		Ref		Ref	
*Above median*	-		**5.85**	**2.16, 15.86**	**5.95**	**1.80, 19.70**
28 week ACTIVE						
*Below median*	-		-		Ref	
*Above median*	-		-		**4.61**	**1.39, 15.24**

Statistically significant relationships (p < 0.05) are in **bold.**

‘ACTIVE’ defined as ≥100 cpm; Median %ACTIVE at T0 = 26.8%; at T1 = 24.7%; at T2 = 26.3%.

T0 = 16^+0^ – 18^+6^ weeks’ gestation; T1 = 27^+0^ – 28^+6^ weeks’ gestation; T2 = 35^+0^ – 36^+6^ weeks’ gestation.

A recording greater than median %ACTIVE at T0 was also strongly associated with a greater than median reduction in %ACTIVE (T1: OR 4.0; 1.53, 10.46; T2: OR 4.16; 1.31, 13.17) (Table 4). There was a strong negative correlation between %ACTIVE at T0 and change in %ACTIVE at T1 (−0.52, p < 0.001) and T2 (−0.56, p < 0.001).

**Table 4:** **Unadjusted odds ratio of reducing ACTIVE as proportion of accelerometer wear time by more than median at T1 and T2 by baseline characteristics**

	**OR**	**95% CI**	**OR**	**95% CI**
Intervention status				
*Intervention*	Ref		Ref	
*Control*	0.44	0.18, 1.12	1.57	0.53, 5.60
Body Mass Index (Kg/m^2^)				
*30-34.9*	Ref		Ref	
*35+*	1.25	0.49, 3.18	2.33	0.77, 7.09
Age (years)				
*<35*	Ref		Ref	
*≥35*	1.58	0.59, 4.27	1.38	0.54, 4.17
Parity				
*Nulliparous*	Ref		Ref	
*Parous*	1.17	0.45, 3.04	0.60	0.19, 1.90
Smoking status				
*Current or ex-smoker*	1.37	0.54, 3.48	1.38	0.45, 4.17
*Never smoked*	Ref		Ref	
Ethnicity				
*White*	Ref		Ref	
*Black, Asian or other*	1.48	0.57, 3.88	1.90	0.62, 5.83
Marital status				
*Married/cohabiting*	Ref		Ref	
*Single/divorced/separate*	0.47	0.18, 1.18	0.40	0.14, 1.21
Educational achievement				
*Degree or higher*	Ref		Ref	
*No degree*	1.94	0.77, 4.90	2.47	0.83, 7.39
Employment status				
*Paid or self employment*	Ref		Ref	
*Not in paid employment*	0.51	0.19, 1.38	1.00	0.30, 3.38
Living accommodation				
*Owned or private rented*	Ref		Ref	
*Rented (council)*	2.38	0.90, 6.27	1.96	0.62, 6.22
IMD quintile				
*1-4*	Ref		Ref	
5 *(most deprived)*	0.73	0.27, 2.00	1.39	0.42, 4.60
Baseline ACTIVE				
*Below median*	Ref		Ref	
*Above median*	**4.00**	**1.53, 10.46**	**4.16**	**1.31, 13.17**
28 week ACTIVE				
*Below median*	-		Ref	
*Above median*	-		2.84	0.91, 8.86

Statistically significant relationships (p < 0.05) are in **bold.**

(median reduction in %ACTIVE was 1.5% at T1 and 0.5% at T2).

T0 = 16^+0^ – 18^+6^ weeks’ gestation; T1 = 27^+0^ – 28^+6^ weeks’ gestation; T2 = 35^+0^ – 36^+6^ weeks’ gestation.

## Discussion

Objectively measured MVPA, but not light or total activity, decreased with gestation in this cohort of obese pregnant women. Our finding is consistent with previous studies of objectively measured PA in non-obese pregnant women [6], and with previous cross-sectional pedometer studies showing lower activity at later gestation in obese pregnant women [10]. This might be attributable to difficulties in maintaining PA as physical discomfort increases as pregnancy progresses [14].

Results from a number of recent trials of lifestyle interventions in overweight and obese pregnant women have demonstrated positive effects. For example, in the TOP (Treatment of Obese Pregnant women) study of 425 obese pregnant women in Denmark, gestational weight gain was lower in women randomised to receive a physical activity intervention, either with or without a dietary component, than in those receiving standard care [20]. In the LIMIT RCT of a combined diet and physical activity lifestyle advice intervention in 2212 overweight and obese pregnant Australian women, fewer macrocosmic infants were born to women randomised to receive lifestyle advice than to those receiving standard care [21]. These findings reinforce the importance of identifying ways of supporting obese pregnant women to make healthy lifestyle choices. However, the intervention in the UPBEAT pilot trial did not have an impact on objectively measured PA. Other trials have reported similar findings. For example, the Fitfor2 study reported no effect of a supervised PA intervention in overweight pregnant women on objectively measured PA [16]. The UPBEAT intervention focused on walking to increase PA. It is possible that in the pilot trial the need to walk at an appropriate (i.e. moderate) intensity was not emphasised sufficiently; women in the intervention group in the UPBEAT pilot trial self-reported an increase in light physical activity [14].

We examined total activity as it has been found to be more strongly associated with insulin sensitivity during pregnancy than sub-components of PA and may be the most appropriate target for interventions to improve glucose tolerance [19]. Baseline activity was the strongest predictor of PA throughout pregnancy. Despite a greater reduction in PA in women who were more active at baseline, this group of women still had a substantially increased likelihood of remaining active throughout pregnancy.

Although this is the first study to consider factors associated with change in objectively measured PA during pregnancy in a cohort of obese women, it has several limitations.

Only 20% of eligible women participated in the UPBEAT pilot trial, raising the possibility of selection bias and attrition was high with less than 40% of women providing data at all time points. However, as women who dropped out of the study did not differ in terms of demographic factors from those who remained in the study, it is unlikely that this affected the findings. The small sample size means type II errors are possible. Accelerometers underestimate upper body activities and cannot capture water-based activities [22]. However accelerometry remains a useful way of measuring PA during pregnancy and in particular intra-individual change [8].

We aimed to identify factors associated with low levels of PA that could help clinicians identify women most likely to benefit from intervention. PA at baseline was the factor most strongly associated with PA during pregnancy. This suggests that an objective assessment of PA in early pregnancy and intervention to support women with low PA to increase their PA and to encourage active women to maintain their PA is warranted. Pre-pregnancy PA has previously been identified as a predictor of PA during pregnancy and appears to be strongly associated with lower risk of GDM [3,23,24]. Population level interventions to encourage all women to be sufficiently physically active irrespective of pregnancy are clearly important.

Systematic review evidence demonstrates that goal setting, self-monitoring and feedback are important in achieving lifestyle behaviour change in pregnant and non-pregnant populations [25,26] and previous work suggests that obese pregnant women specifically require active involvement in setting individualised goals and intensive support and feedback to make behaviour changes [11,25]. Previous qualitative work in the UK found obese pregnant women feel they do not receive adequate advice and support from health care providers around appropriate PA during pregnancy and would welcome more guidance [27]. Similar findings have been reported from the US [28]. Pain during pregnancy can be severe enough to impact on usual daily activities [19]. These findings present several modifiable barriers to PA that could be addressed in the development of interventions to support women to be active during pregnancy. Interventions should include appropriate advice on the benefits of PA and support to set and monitor PA goals from health care professionals and advice on coping with pregnancy-related pain.

## Conclusion

Identifying ways of supporting women to be sufficiently active during pregnancy remains a challenge. The clearest predictor of change in PA during pregnancy in this study was level of PA at baseline. Women who were most active at the beginning of their pregnancy maintained their activity level better than those who were less active. This indicates the importance of emphasising the health benefits associated with physical activity to obese women within pre-conception planning when the opportunity arises, and at booking appointments.
